# Characterizing the *in vitro* and *in vivo* effect of bicarbonate on azithromycin activity against *Acinetobacter baumannii*

**DOI:** 10.1371/journal.pone.0350230

**Published:** 2026-06-03

**Authors:** Hadley Jaramillo, Matthew Slarve, Derek Long, Michelle Zorawik, Kristine Goy, Lauren Popel, Rosemary She, Brad Spellberg, Brian Luna

**Affiliations:** 1 Department of Immunology and Immune Therapeutics, Keck School of Medicine at USC, Los Angeles, California, United States of America; 2 Department of Pathology, City of Hope, Duarte, California, United States of America; 3 Los Angeles General Medical Center, Los Angeles, California, United States of America; Università degli Studi Roma Tre Dipartimento di Scienze: Universita degli Studi Roma Tre Dipartimento di Scienze, ITALY

## Abstract

Carbapenem-resistant *Acinetobacter baumannii* (CRAB), a Gram-negative bacterial pathogen, has been identified by Centers for Disease Control (CDC) as the top priority pathogen for which new antibiotics are needed. We found that the addition of bicarbonate at physiologically normal levels found in the blood (23.8 mM) increased susceptibility of *A. baumannii* clinical isolates (n = 63) to azithromycin and resulted in MIC50 shift from 64 mg/L in CAMHB to 1–2 mg/L in CAMHB + bicarbonate (23.8 mM) or RPMI-1640 respectively. To characterize *in vivo* efficacy in murine blood and lung infection models, mice were infected with *A. baumannii* and then mice were treated with a human equivalent dosing strategy of azithromycin. *In vivo* outcomes greatly depended on the infection model used. The bloodstream infection model showed a statistically significant increase in survival of the treatment group compared to the control group. However, that was not found with the oral aspiration infection model. We hypothesize that these *in vivo* results are due to the local differences of bicarbonate concentrations at the site of infection throughout the course of infection.

## Introduction

Many antibiotic-resistant infections still remain susceptible to older antibiotics that are inexpensively available as generics. Due to massive differences in cost, but not necessarily health-related outcomes, it remains important to ensure that our inventory of antibiotics is being used as efficiently as possible. Azithromycin (AZM) is the second most frequently prescribed antibiotic in the US, has a broad spectrum of activity, and has favorable safety characteristics [1,2]. Macrolides are traditionally thought active against atypical pneumonia agents, [3] but not against non-fermenting Gram-negative pathogens such as *A. baumannii.* Therefore, no clinical laboratory standards exist for evaluating AZM activity against *A. baumannii* [4]. To the best of our knowledge, there is no clinical data that describe AZM monotherapy for the treatment of *A. baumannii* infection. However, a case study reported that the combination of AZM+meropenem to be effective for the treatment of combat injuries in the Ukraine war that included MDR- and XDR- *A. baumannii* isolates [5].

Our group previously characterized rifabutin’s iron-dependent efficacy against *A. baumannii* using more physiologically relevant RPMI-1640 mammalian culture medium, which does contain physiologically normal concentration of bicarbonate, to perform AST [6,7]. AZM’s mechanism of entry into bacterial cells depends on alteration of transmembrane proton motive force by bicarbonate, which is present in the host environment and RPMI-1640 but absent in CAMHB medium [8,9]. Our lab and other independent labs have characterized examples where *in vitro* susceptibility testing in bicarbonate-containing medium had better predicted *in vivo* outcomes [8–16]. We therefore evaluated AZM activity against carbapenem-resistant *A. baumannii* clinical isolates in RPMI-1640 versus CAMHB media with and without bicarbonate.

Furthermore, we recently found that the nonconventional RPMI-1640 medium, which is relatively nutrient-depleted and therefore more physiologically relevant than traditional rich media, for antibiotic susceptibility testing better predicted treatment outcomes *in vivo* for some antibiotics [6,7,13,14,17,18]. Therefore, we characterized the antimicrobial effects of AZM *in vitro* by determining its MICs for *A. baumannii* using both conventional CAMHB, CAMHB supplemented with bicarbonate, or RPMI-1640.

We previously found that AZM treatment outcomes in a *Galleria mellonella* infection model were better predicted by *in vitro* susceptibility testing conducted with the RPMI-1640 medium, and not the standard CAMHB medium [15]. These data suggest that AZM may have promise as a novel therapy for highly resistant *A. baumannii,* and that traditional susceptibility testing may not accurately predict this potential *in vivo* benefit. A limitation of the *Galleria mellonella* infection model is that AZM pharmacokinetics are not well described in this host. To better support clinical translation, we evaluated AZM efficacy in clinically relevant murine infection models using a human equivalent dosing strategy for AZM.

## Materials and methods

### Ethics statement

All animal work was conducted following approval by the Institutional Animal Care and Use Committee (IACUC protocol 21557) at the University of Southern California, in compliance with the recommendations in the Guide for the Care and Use of Laboratory Animals of the National Institutes of Health. Infected mice develop weight loss, ruffled fur, poor appetite, decreased ambulation, huddling behavior, and low body temperature. Mice that display huddling behavior and are poorly mobile will be weighed 1x daily. Weight loss of greater than 15% body weight will trigger euthanasia. Mice were monitored at least twice daily for seven days. Soft bedding and other enrichment devices were provided as recommended by the veterinary staff. Nutritional supplements, such as the hydrogel packs were provided as needed. Mice were not found dead prior to euthanasia. At study endpoints, mice were anaesthetized by IP administration of ketamine (100 mg/kg) + xylazine (10 mg/kg) and a terminal cardiac puncture was performed.

### Bacteria and culture

Fifty contemporary carbapenem-resistant clinical isolates, collected between 2020–2023 were obtained from the ARUP Laboratories, a national reference clinical laboratory. An additional 13 isolates that have been previously characterized in murine infection models were also included. Overnight cultures of *A. baumannii* were grown in Tryptic Soy Broth (TSB) at 37°C. The overnight culture was diluted 1:100 and then subcultured in CAMHB at 37°C/ 200 rpm until the culture reached an OD600 of 0.5. The log-phase culture was diluted to the target inoculum prior to infection. The inoculum was determined by plating serial dilutions on TSA plates.

### MIC protocol

MIC assays were done by broth microdilution according to standard CLSI protocols or modified by supplementing with a range of sodium bicarbonate from 0 to 2 g/L as specified in the results or substituting with RPMI-1640 with 10% or 20% FBS as indicated in the table or figure legend. Briefly, using the colony suspension method, after 18–24 hour growth on non-selective agar, 3–5 isolated colonies were suspended in saline and then adjusted to achieve a 0.5 McFarland solution. The organism suspension was added to wells of a 96-well plate containing serial drug dilutions in culture media (culture media alone or drug media) to a final concentration of 5 x 10^5^ CFU/mL. MICs were determined using the broth microdilution method covering a range of 2-fold serial dilutions from 0.125 to 64 mg/L. MICs were recorded after plate incubation at 35 ± 2°C for 20–24 hr in ambient air.

### Antibiotic preparation

A fresh stock of AZM (Slate Run Pharmaceuticals, NDC 70436-019-82) was prepared daily for the MIC assay. For *in vitro* testing, the stock solution was prepared by dissolving the drugs in molecular grade sterile water. The working solution of antibiotic was prepared 2X of the desired test drug concentration.

### Mouse studies

Healthy male and female, immune normal C3HeB/FeJ (Jackson Laboratory, stock no. 000658) mice were used, as we and others have previously found that such mice are more susceptible to infection caused by *A. baumannii* than other commonly used inbred strains [19].

### Intravenous (IV) infection

Bacterial inoculum, made from fresh or frozen bacteria, was prepared as described in previous work [20]. 8-week old male and 10-weeks old female C3HeB/FeJ mice were infected via tail vein injection and the inoculum bacterial density was confirmed by plating serial dilutions on TSA plates and incubated overnight at 37°C.

### Oral aspiration (OA) infection

Bacterial inoculum, made from fresh or frozen bacteria, was prepared as described in previous work [20]. 8-week old male and 10-weeks old female C3HeB/FeJ mice were infected via an oral aspiration infection procedure as previously described [21]. The inoculum bacterial density was confirmed by plating serial dilutions on TSA plates and incubating overnight at 37°C.

### Quantification of *in vivo* CFUs

For the blood infection model, at 18 h post infection mice were anesthetized by ketamine (100 mg/kg/IP) and xylazine (10 mg/kg/IP) and then euthanized, and blood was collected by terminal cardiac puncture and transferred to heparinized tubes. For the OA infection model, lungs were harvested and homogenized. Serial dilutions of blood or lung homogenate were plated on TSA plates and incubated overnight at 37˚C.

### AZM treatment

Mice were treated with a humanized equivalent dosing strategy of AZM as previously described [22]. At 2 hours post infection, mice were treated s.c. with 10.5 mg/kg/daily AZM which corresponds to the human equivalent dosing of 500 mg AZM in humans or PBS as a control [22].

### Statistical analysis

Survival was compared by the nonparametric log-rank test. Bacterial density was compared with the Mann-Whitney test for unpaired comparisons. All statistics were calculated using R software. Differences were considered significant if the P-value was < 0.05.

## Results

**Fig 1:**
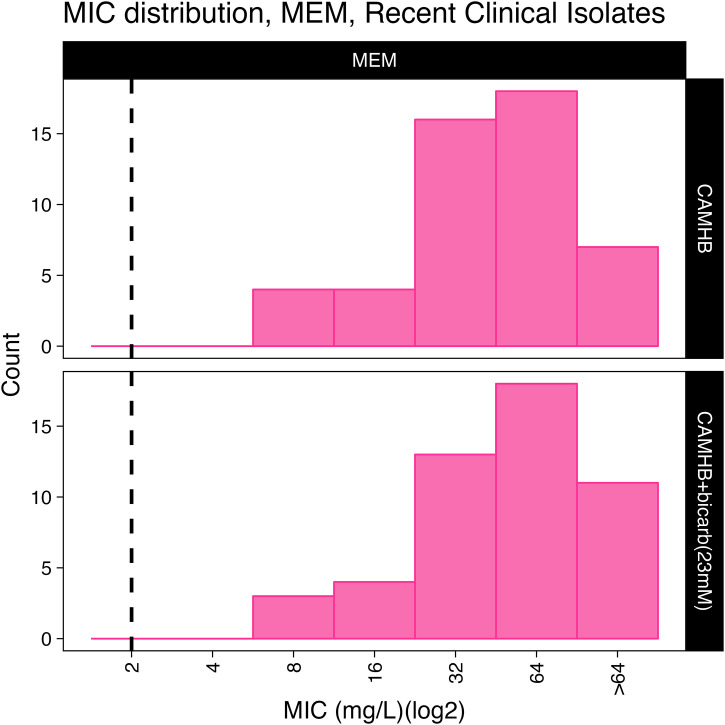
MEM MICs for *A. baumannii* clinical isolates as determined in CAMHB or CAMHB+bicarbonate media. The distribution of MICs for a panel of *A. baumannii* clinical isolates is represented as a histogram. The dashed black line corresponds to the MEM resistant breakpoint of ≥8 mg/L for MEM.

MICs were determined against a panel of 63 clinical isolates. This panel consisted of 50 contemporary carbapenem-resistant clinical isolates collected from 2020–2023 and 13 isolates that have been previously characterized in murine infection models. Meropenem (MEM) MICs were measured to confirm that all isolates were carbapenem-resistant (Fig 1**).**

**Fig 2:**
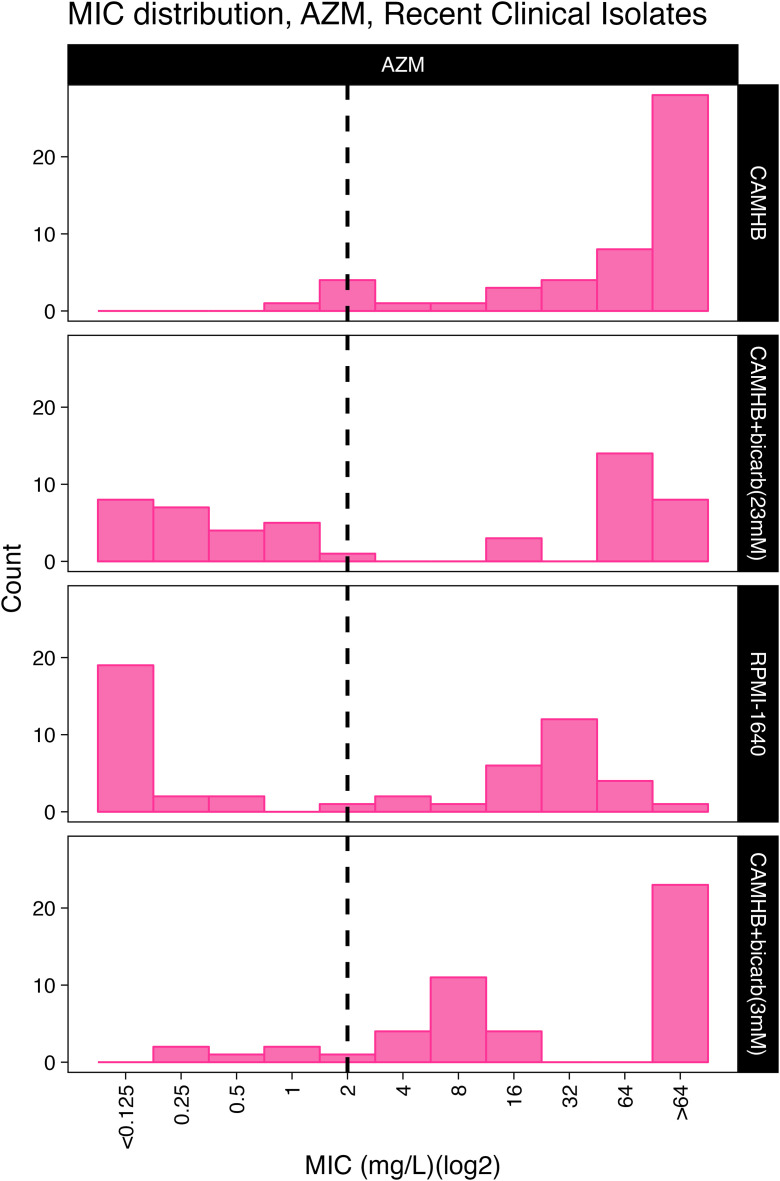
AZM MICs for *A. baumannii* clinical isolates as determined in CAMHB. **CAMHB+bicarbonate media, or RPMI-1640 + 10% FBS.** The distribution of MICs for a panel of *A. baumannii* clinical isolates is represented as a histogram. The dashed black line corresponds to the AZM resistant breakpoint of 2 mg/L for AZM. Because AZM breakpoints are not available for *A. baumannii*, we used the CLSI breakpoints for *S. aureus.* Dashed lines indicated the cutoff for the resistant breakpoint interpretation.

**Fig 3:**
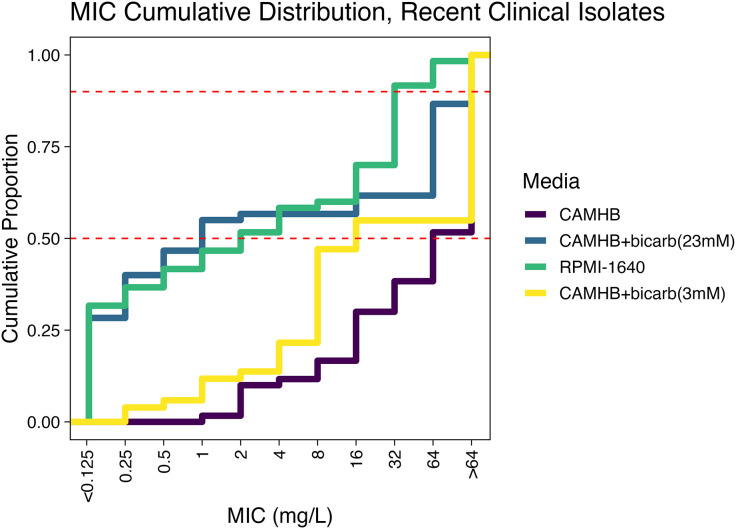
AZM MICs for *A. baumannii* clinical isolates (n = 63) as determined in CAMHB. CAMHB+bicarbonate media, or RPMI-1640 + 10% FBS. The cumulative distribution of MICs for a panel of *A. baumannii* clinical isolates. The dashed red lines correspond to the MIC_50_ and MIC_90_ respectively.

**Fig 4:**
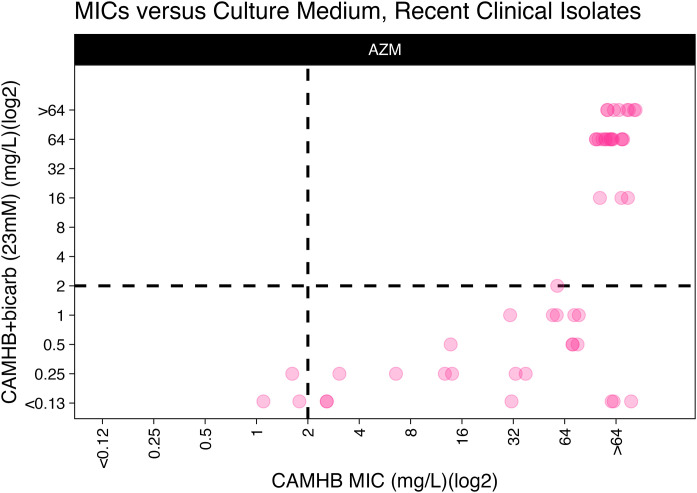
AZM MICs for *A. baumannii* clinical isolates (n = 63) as determined in CAMHB or CAMHB+bicarbonate (23mM) media. The lower right quadrant represents isolates that would be predicted to be resistant in CAMHB but susceptible when tested in the 23 mM bicarbonate media condition. The MICs for each isolate are represented as a light gray circle, and dark gray to black circles indicate overlapping isolates.

*A. baumannii* is largely resistant to AZM when MICs are determined using standard testing conditions with CAMHB media (Table 1, Figs 2–4). However, we found that the addition of bicarbonate at physiologically normal levels found in the blood (23 mM) would enhance susceptibility of the *A. baumannii* to AZM and resulted in MIC_50_ shift from 64 mg/L in CAMHB to 1 mg/L or 2 mg/L in CAMHB + bicarbonate (23.8 mM) or RPMI-1640 respectively. There was a significant difference in the distribution of MICs determined in CAMHB as compared to CAMHB+bicarbonate (23.8mM) (Mann-Whitney, p = 8.58e-13) or RPMI-1640 (Mann-Whitney, p = 7.32e-20). There was no significant difference between CAMHB + bicarbonate (23.8 mM) and RPMI-1640. Lastly, we found that reducing the concentration of bicarbonate in the media to 3 mM resulted in a MIC_50_ = 16 mg/L and the isolates appeared mostly resistant to AZM (Table 1, Fig 2-3).

**Table 1:** AZM MIC_50_ and MIC_90_ percentile values for n = 63 clinical isolates.

Media	MIC_50_ (mg/L)	MIC_90_ (mg/L)
**CAMHB**	64	>64
**CAMHB +** **Bicarbonate (23.8 mM)**	1	>64
**CAMHB +** **Bicarbonate (3 mM)**	16	>64
**RPMI-1640 + 10% FBS**	2	32

**Fig 5:**
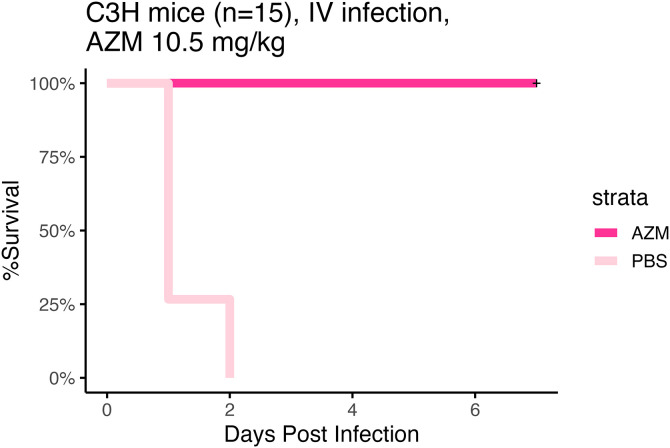
*In vivo* efficacy of AZM treatment, time to moribund endpoint. C3HeB/FeJ mice (n = 10 males + n = 5 females per treatment group) were infected with a lethal inoculum of *A. baumannii* strain VA-AB41. Mice were infected by tail vein IV injection method and mice were treated with PBS as a control or a human equivalent dosing strategy of AZM (10.5 mg/kg) beginning at 2 h post infection. A significant difference was observed between the PBS and AZM groups (Log-Rank, p = 1.7E-8).

To evaluate if the MICs as determined in the presence of bicarbonate were more predictive of *in vivo* outcome, we first evaluated AZM efficacy in a bloodstream infection model (Fig 5). Mice were infected with the clinical isolate *A. baumannii* VA-AB41 that appeared susceptible in media containing bicarbonate but was resistant in standard CAMHB media. There was a significant difference between AZM treated mice and the PBS control (Log-Rank test, p = 1.8E-8) (Fig 5).

Despite the partial success that was observed, we then opted to test efficacy in an oral aspiration pneumonia model because meta-analysis of extensive data has found that macrolide resistance *in vitro* does not predict clinical failure when macrolides are used to treat pneumonia caused by *Streptococcus pneumoniae* [23]. However, substantially higher failure rates have been observed when macrolides are used to treat bacteremia (including bacteremic pneumonia) caused by *S. pneumoniae* that are susceptible or resistant to macrolides [23].

Prior to initiating AZM treatment efficacy experiments, we first needed to characterize the virulence of the clinical isolates to be used in the oral aspiration pneumonia model. We found modest differences in the inoculums needed to produce lethal infections in male and female mice, with the notable exception that female mice require about 20x more VA-AB41 CFUs to produce a lethal infection as compared to male mice (Table 2). This difference in the required inoculum is consistent with past work as well [20].

**Table 2:** *In vivo* virulence characterization *A. baumannii* clinical isolates in C3HeB/FeJ mice. Mice (n = 5 male and n = 5 female mice) were used to define lethal and sublethal inoculums in an OA infection model.

	Female		Male	
Strain	Sub-LD100 (CFU/mouse)	LD100 (CFU/mouse)	Sub-LD100 (CFU/mouse)	LD100 (CFU/mouse)
**1057039**	4.00E + 08	5.00E + 08	4.00E + 08	5.00E + 08
**LAC-4**	2.50E + 07	3.00E + 07	2.50E + 07	3.00E + 07
**1112707**	8.20E + 07	2.40E + 08	2.40E + 08	3.00E + 08
**VA-AB41**	3.50E + 08	4.50E + 08	1.40E + 07	2.00E + 07
**112747**	3.00E + 08	5.00E + 08	1.89E + 08	3.00E + 08
**HUMC1**	2.50E + 08	5.00E + 08	2.50E + 08	5.00E + 08

In healthy animals, the concentration of bicarbonate is different in the blood compartment (23.8 mM) and the epithelial lining fluid (ELF) of the lung (11 mM) [24]. We therefore determined AZM MICs across a range of bicarbonate conditions with the understanding that bicarbonate concentration in the ELF should decrease as the mice become acidotic during infection. We found that reducing the amount of bicarbonate to 3 mM resulted in a MIC shift from a susceptible to resistant breakpoint interpretation (Table 3). The changes in MIC would also change the estimated AUC/MIC ratio for infected mice (Table 4**).** For AZM efficacy, an AUC/MIC ratio of 25–35 is required [25].

**Table 3:** AZM MICs against virulent *A. baumannii* clinical isolates that were used for *in vivo* infection models. CAMHB media was supplemented with various concentrations of bicarbonate as described below. RPMI-1640 was supplemented with 20% FBS by volume.

Strain	AZM MIC (mg/L)								
	RPMI- 1640	CAMHB + Bicarbonate							
		23.8 mM	20 mM	15 mM	10 mM	3 mM	1 mM	0.3 mM	0 mM
1057039	0.13	0.13	1	1	2	16	16	32	32
LAC-4	0.13	0.13	0.13	0.5	0.25	1	1	2	>64
1112707	0.13	0.13	0.25	0.25	0.5	8	32	32	16
VA-AB41	0.13	0.13	0.13	0.25	0.50	8	16	32	16
112747	0.13	0.13	1	0.50	1	8	32	32	16
HUMC1	32	>64	>64	>64	>64	>64	>64	>64	>64

**Table 4:** AZM AUC/MIC ratios against virulent *A. baumannii* clinical isolates used for *in vivo* infection models. Ratios were calculated based on a AZM AUC of 10.56 mg*h/L [22].

AZM AUC/MIC Ratio				
		Supplemented bicarbonate		
**Strain**		**23.8 mM**	**10 mM**	**3 mM**
**1057039**		81.23	5.28	0.66
**LAC-4**		81.23	42.24	10.56
**1112707**		81.23	21.12	1.32
**VA-AB41**		81.23	21.12	1.32
**112747**		81.23	10.56	1.32
**HUMC1**		0.0825	0.0825	0.0825

**Fig 6:**
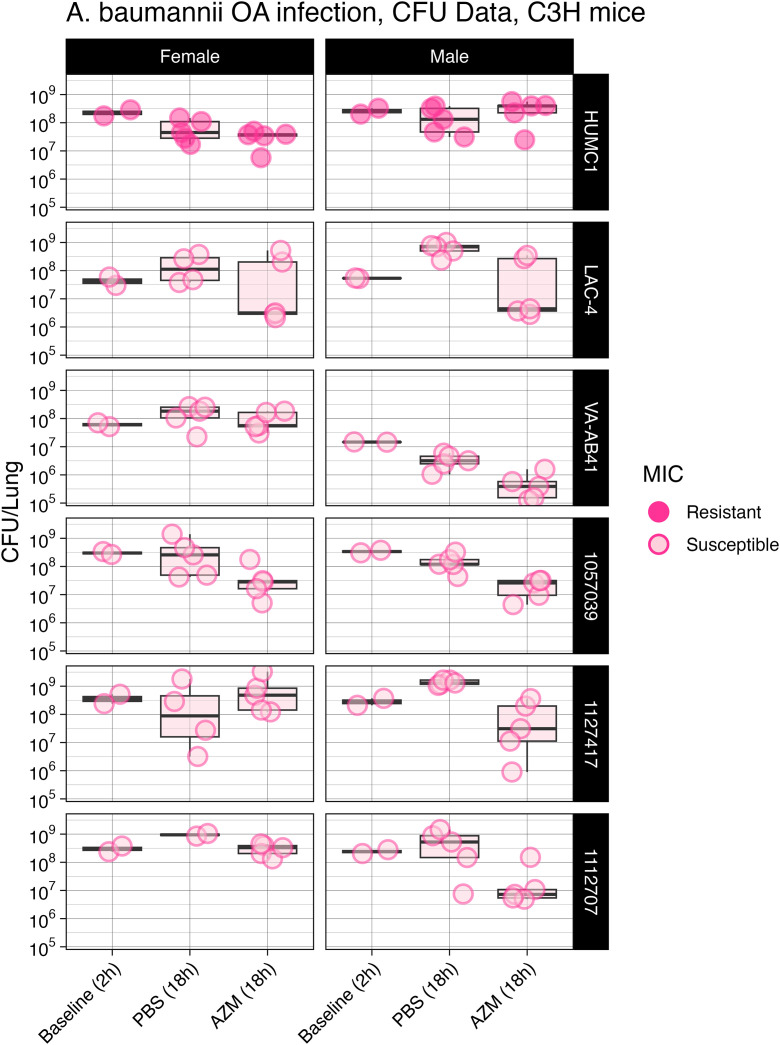
*In vivo* efficacy of AZM treatment, microbiology endpoint. C3HeB/FeJ mice (n = 5 males + n = 5 females) were infected with a lethal inoculum of *A. baumannii* strain HUMC1, LAC-4, VA-AB41, 1057039, 112747, or 112707. Mice were infected by OA method and lungs were homogenized and serial dilutions were plated to determine CFUs. Two mice were sacrificed at 2 h post infection to establish our baseline CFUs after infection and immediately prior to treatment. Mice were treated with PBS as a control or a human equivalent dosing strategy of AZM beginning at 2 h post infection. A significant difference was observed between the PBS and AZM groups at 18 h in male mice for strains LAC-4 (Mann-Whitney, p = .017), 1057039 (Mann-Whitney, p = 0.025) and 112747 (Mann-Whitney, p = 0.0003). Strains with MICs in 23.8 mM bicarbonate containing media < 2 mg/L or >2 mg/L were categorized as “susceptible” or “resistant” respectively.

**Fig 7:**
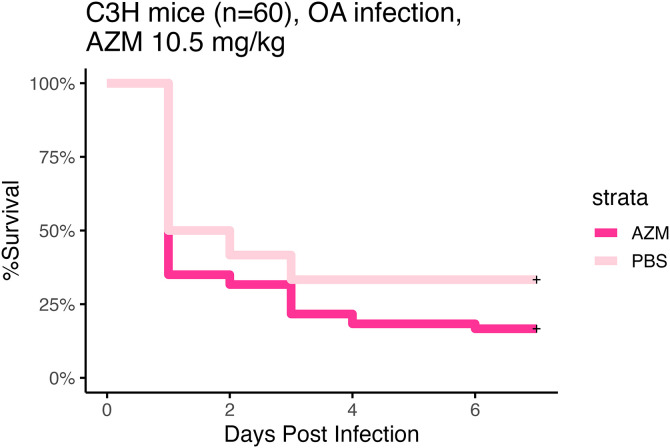
*In vivo* efficacy of AZM treatment, time to moribund endpoint. Treatment groups (n = 30 male + n = 30 female mice per group) for all infecting strains were combined and overall survival curves were plotted. There was a significant difference between treatment groups (Log-Rank, p = 0.049).

C3HeB/FeJ mice were challenged with the *A. baumannii* strains shown in Table 2 and we measured the effect of AZM treatment on changes to lung CFUs. A significant difference was observed between the PBS and AZM groups at 18 h in male mice for strains LAC-4 (Mann-Whitney, p = .017), 1057039 (Mann-Whitney, p = 0.025) and 1127417 (Mann-Whitney, p = 0.0003) (Fig 6). Consistent with the apparent lack of treatment benefit in the CFU studies, treatment with AZM did not improve survival as compared to the PBS control group (Fig 7**).**

## Discussion

Consistent with previous publications from our group and other independent groups, we found that the addition of a physiologically relevant concentrations of bicarbonate would affect the susceptibility of Gram-negative bacteria to AZM [8,9,13,15,26]. One possible limitation of our *in vitro* testing is that our model would not be able to estimate possible potentiation of bicarbonate, AZM, and the innate immune system that has been previously described [13,27]. We found that higher concentrations of bicarbonate result in the bacteria becoming more susceptible to AZM and that this effect decreases when the concentration of bicarbonate is reduced (Table 1,3). The use of media supplemented with 23.8 mM bicarbonate, a concentration that is in the physiological normal range of blood in healthy mice and humans, resulted in the lowest MICs.

The MIC data look most promising when testing in media that mimic the bicarbonate concentration in the blood. However, AZM looks less effective when MICs are conducted in media that mimic bicarbonate concentrations found in the ELF. We observed higher MICs at 10 mM bicarbonate condition, which is the normal bicarbonate concentration in the ELF of healthy mice. However, we anticipate that the concentration of bicarbonate in the ELF will decrease in sick animals as the free bicarbonate will be neutralized by the acidic environment that is characteristic of bacterial infections. MICs at the 3 mM bicarbonate condition represent a shift in MIC_50_ from a susceptible to resistant breakpoint interpretation (Tables 1–3).

We were able to identify virulent clinical isolates that could be tested in immunocompetent mice (Table 2). All isolates in the panel were predicted to be resistant based on standard MIC testing using CAMHB. However, five of the six isolates were predicted to be susceptible when MICs were done using the 23.8 mM or 10 mM bicarbonate conditions (Table 3). However, we generally did not observe treatment benefits for any of the isolates tested. A significant reduction in CFUs was only observed for three of six infecting strains, but the benefit was only observed in male mice (Fig 4). For these strains, the LD100 values were very similar for both genders, so the differences in treatment responses based on gender is not due to disparities in administered inoculums (Table 2).

Consistent with the lack of CFU reductions in response to treatment, we did not observe a survival benefit in the OA infection model (Fig 7**).** The CFU and survival outcomes appear to be consistent with decreased activity of AZM at lower concentrations of bicarbonate (Table 3**).** The lack of a survival benefit was consistent with a previous published study from an independent group [13]. Future research is needed to empirically determine the local concentration of bicarbonate in the ELF to better optimize antibiotic susceptibility testing for the specific site of infection.
